# Inflammation and acute cardiotoxicity in adult hematological patients treated with CAR-T cells: results from a pilot proof-of-concept study

**DOI:** 10.1186/s40959-024-00218-0

**Published:** 2024-03-27

**Authors:** Massimiliano Camilli, Marcello Viscovo, Tamara Felici, Luca Maggio, Federico Ballacci, Giacomo Carella, Alice Bonanni, Priscilla Lamendola, Lorenzo Tinti, Antonio Di Renzo, Giulia Coarelli, Eugenio Galli, Giovanna Liuzzo, Francesco Burzotta, Rocco Antonio Montone, Federica Sorà, Simona Sica, Stefan Hohaus, Gaetano Antonio Lanza, Filippo Crea, Antonella Lombardo, Giorgio Minotti

**Affiliations:** 1 Department of Cardiovascular and Pulmonary Sciences, Catholic University of the Sacred Heart, Rome, Italy; 2 Department of Cardiovascular Medicine, Fondazione Policlinico Universitario A. Gemelli IRCCS, Rome, Italy; 3 Sezione di Ematologia, Dipartimento di Scienze Radiologiche ed Ematologiche, Università Cattolica del Sacro Cuore, Rome, Italy; 4 Dipartimento di Diagnostica per Immagini, Radioterapia Oncologica ed Ematologia, Fondazione Policlinico Universitario A. Gemelli IRCCS, Rome, Italy; 5 University Campus Bio-Medico, Rome, Italy; 6 Università e Fondazione Policlinico Universitario Campus Bio-Medico, Rome, Italy

**Keywords:** Chimeric antigen receptor-T cells, Inflammation, Cardiotoxicity, Echocardiography, Cardio-Oncology, Hematological malignancies

## Abstract

**Aims:**

Chimeric Antigen Receptor-T (CAR-T) cell infusion is a rapidly evolving antitumor therapy; however, cardiovascular (CV) complications, likely associated with cytokine release syndrome (CRS) and systemic inflammation, have been reported to occur. The CARdio-Tox study aimed at elucidating incidence and determinants of cardiotoxicity related to CAR-T cell therapy.

**Methods:**

Patients with blood malignancies candidate to CAR-T cells were prospectively evaluated by echocardiography at baseline and 7 and 30 days after infusion. The study endpoints were i) incidence of cancer therapy-related cardiac dysfunction (CTRCD), CTRCD were also balanced for any grade CRS, but CTRCD occurred of Cardiology Guidelines on Cardio-Oncology (decrements of left ventricular ejection fraction (LVEF) or global longitudinal strain (GLS) and/or elevations of cardiac biomarkers (high sensitivity troponin I, natriuretic peptides) and ii), correlations of echocardiographic metrics with inflammatory biomarkers.

**Results:**

Incidence of CTRCD was high at 7 days (59,3%), particularly in subjects with CRS. The integrated definition of CTRCD allowed the identification of the majority of cases (50%). Moreover, early LVEF and GLS decrements were inversely correlated with fibrinogen and interleukin-2 receptor levels (*p* always ≤ 0.01).

**Conclusions:**

There is a high incidence of early CTRCD in patients treated with CAR-T cells, and a link between CTRCD and inflammation can be demonstrated. Dedicated patient monitoring protocols are advised.

**Graphical Abstract:**

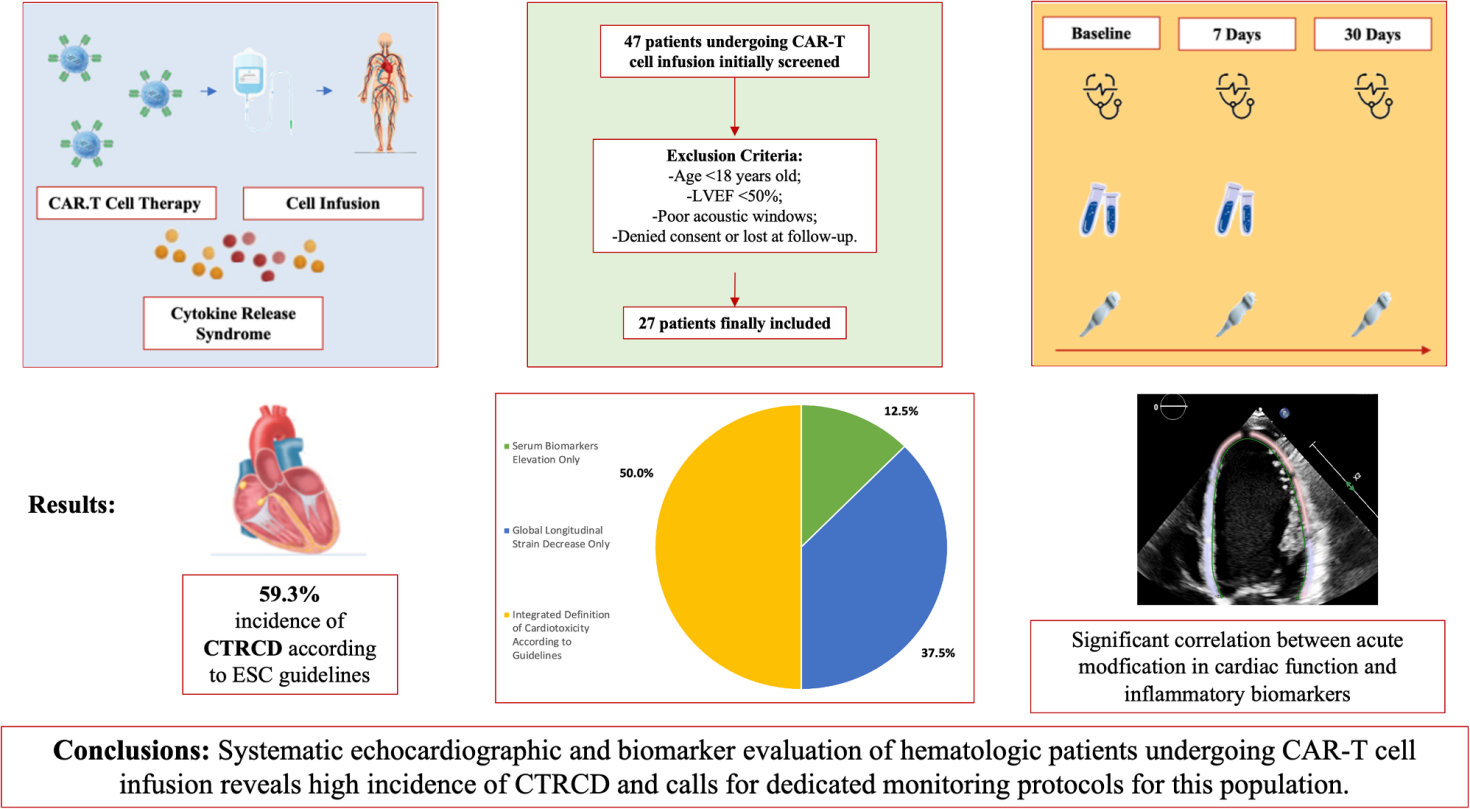

**Supplementary Information:**

The online version contains supplementary material available at 10.1186/s40959-024-00218-0.

## Introduction

Chimeric antigen receptor (CAR)-T cell therapy represents an effective therapeutic opportunity for patients with advanced hematological malignancies, delivering a significant improvement in response rates [1–3]; however, data from clinical trials and real-word reports show that numerous adverse events may occur [4, 5]. The main CAR-T cell toxicity is represented by the cytokine release syndrome (CRS), a subtype of systemic inflammatory response syndrome [6] which is characterized by an excessive systemic inflammatory response secondary to the interaction between the engineered T-cells, immune effectors and the tumor microenvironment [7]. Clinical presentation of CRS ranges from mild flu-like symptoms to life-threatening multiorgan dysfunction [7]. These manifestations do not spare the cardiovascular (CV) system, as denoted by cases of severe cardiac dysfunction, arrhythmias, and cardiovascular death [8–10].

Cause-and-effect relations between CRS severity and CV toxicity have been hypothesized [8–10], but prospective studies that probed optimal modalities for detecting and monitoring inflammation and CV toxicity in this unique patient population are scarce [11]. The CARdio-TOX study is a single center, prospective, proof-of-concept study of adult patients affected by refractory or relapsed (R/R) hematologic malignancies and treated with CAR-T cells. We recently reported that patients recruited in CARdio-TOX exhibited impaired left ventricle ejection fraction (LVEF) and global longitudinal strain (GLS) as early as 7 days after CAR-T cells infusion, with concomitant changes of several exploratory diastolic metrics occurring at the same time point [12]. Patient re-evaluation at 30 days showed an incomplete recovery of systolic and diastolic parameters, possibly denoting that acute myocardial damage may or may not resolve over time and pave the road to chronic toxicity and late clinical outcomes.

In the present study, we aimed at elucidating both the incidence of Cancer therapy-related cardiac dysfunction (CTRCD), defined as an aggregate of echocardiographic and biomarker abnormalities, and correlations between imaging alterations and bio-humoral indexes of inflammation.

## Patients and methods

### Study design and patient characteristics

CARdio-TOX is a non-profit, investigator-initiated, prospective, single center, real-life study conducted at the Department of Cardiovascular Medicine of Fondazione Policlinico Universitario A. Gemelli IRCCS, Rome, Italy, between April 2022 and April 2023. Adult patients eligible to CAR-T cell therapy underwent clinical, electrocardiographic and echocardiographic evaluations at baseline and then at 7 and 30 days after CAR-T cell administration. Three anti CD19 CAR-T cell preparations were used according to clinicians’ indications: Axicabtagene Ciloleucel (Yescarta, Kite Pharmaceuticals, Santa Monica, California), Tisagenlecleucel (Kymriah, Novartis Pharmaceuticals, East Hanover, New Jersey), Brexucabtagene Autoleucel (Tecartus, Kite Pharma EU B.V.). Inclusion criteria were age > 18, years, LVEF ≥ 50% and a confirmed diagnosis of R/R CD19^+^ B-cell malignancy (lymphoma or acute lymphoblastic leukemia) with two or more prior systemic therapies. Exclusion criteria were age < 18 years and left ventricular ejection fraction (LVEF) < 50% or poor acoustic window at baseline echocardiography. The following information was extracted from patient medical records: prior CV events, CV risk factors (arterial hypertension, smoking, diabetes mellitus, dyslipidemia), history of potentially cardiotoxic therapies (chemotherapy, immunotherapy, left chest radiation therapy, autologous hematopoietic stem cell transplantation [HSCT].

The primary objective was the incidence of CTRCD at 7 days after CAR-T cell infusion. CTRCD was defined according to 2022 European Society of Cardiology (ESC) Cardio-Oncology guidelines (LVEF reduction by ≥ 10% points to an LVEF of 40–49%, or LVEF reduction by < 10% points to an LVEF of 40– 49%, or a decline of global longitudinal strain (GLS) by ≥ 15% from baseline, or increases in cardiac biomarkers such as troponin or B-type natriuretic peptide) [13]. Indexes of LV dysfunction (LVEF, GLS) were then correlated with serum inflammatory biomarkers such as C-reactive protein (CRP), fibrinogen, ferritin, soluble interleukin 2 receptor (sIL-2r), interleukin 6 (IL-6).

The study was approved by the Institutional Ethic Committee. No extramural funding supported this work. The authors are solely responsible for study design and conduct, study analyses, drafting and editing of the paper, as well as its final content.

### Echocardiographic evaluation

Transthoracic 2D echocardiography (TTE) was performed using Philips EPIQ7C (Philips Medical Systems, Andover, Massachusetts, USA). Colour, pulsed-wave and continuous wave Doppler images were acquired from the parasternal, apical and subcostal views [14, 15]. All images were digitally stored for offline analyses by an experienced operator (L.M.). 2D-Strain (2D-ST) analysis was determined from views acquired during three consecutive cardiac cycles, using a TomTec-Arena TM software (TomTec Imaging Systems, Unterschleissheim, Germany). LV-GLS was calculated from the average values of four-chamber, two-chamber, and three-chamber curves. LV dimension, LA volume with strain analysis and right ventricle (RV) longitudinal function were measured according to recommendations by the European Association of Cardiovascular Imaging (EACVI) and the American Society of Echocardiography (ASE) [14–16]. LV diastolic function, non-invasive estimation of LV filling pressures, and valvular heart diseases were evaluated according to current recommendations [17, 18]. Intra-observer and inter-observer variability assessment and values for our echocardiographic laboratory have been previously described [19].

### Cardiac and inflammatory biomarkers

Blood samples for cardiac and inflammatory biomarkers were drawn before and 7 days after CAR-T cell infusion. High-sensitivity troponin I (hsTnI), soluble protein ST2 (sST2) and the aminoterminal fragment of prohormone BNP (Nt-proBNP) were used as cardiac biomarkers; IL6, sIL2r, ferritin and fibrinogen were used as inflammatory biomarkers as per institutional clinical practice. All biomarkers were measured according to validated protocols of the institutional Medicinal Chemistry department. CRS grade and any required treatment for CRS management were in accordance to the American Society for Transplantation and Cellular Therapy (ASTCT) consensus [20].

### Statistical analyses

Dichotomous variables were expressed as counts (percentage). The distribution of continuous variables was tested using Kolmogorov–Smirnov test. Mean ± standard deviation was used to express continuous variables with normal distribution, while median (interquartile range) was used for variables with non-normal distribution. Continuous variables were compared using an unpaired Student’s t test or Mann–Whitney U test. Categorical data were evaluated using the χ2 test or Fisher exact test, as appropriate.

Differences in each continuous echocardiographic parameter and inflammatory biomarker between day 7 and baseline were expressed as ∆:100*(day 7 minus baseline)/baseline.

All tests were two-sided, and statistical significance was set at *P* < 0.05. All analyses were performed using SPSS (SPSS version 23, Inc., Chicago, IL, USA) statistical software.

## Results

### Patient characteristics and CTRCD incidence

**Fig. 1:**
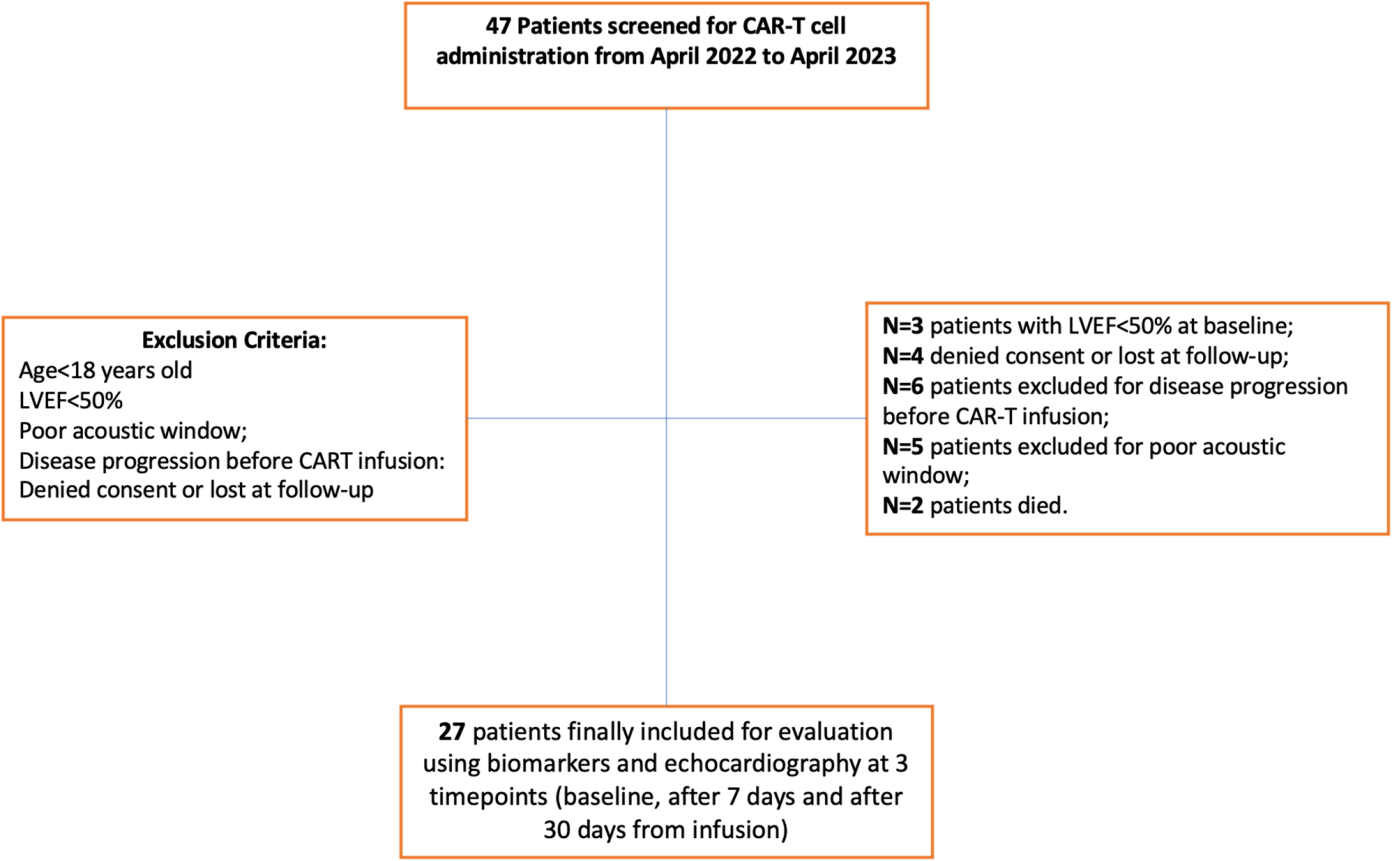
Study Flowchart. Abbreviations CAR-T = Chimeric Antigen Receptor-T; LVEF = left ventricular ejection fraction

Forty-seven patients candidate for CAR-T cell therapy were screened, of whom 27 were eventually enrolled (Fig. 1).Sixteen patients (59,3% of the study population) were diagnosed CTRCD as per guidelines definition [13]. Patients with or without CTRCD were balanced for age, oncologic characteristics, common comorbidities and risk factors (hypertension, diabetes, smoking), as well as baseline laboratory and imaging findings; however, cardiotoxicity occurred more frequently in females (*p* = 0.042). CRS occurred in a total of 24 patients (88.8%) and the anti IL6 receptor antibody, tocilizumab, was used in 19 (70.4%) patients. Patients with or without CTRCD were also balanced for any grade CRS, but CTRCD occurred more often in patients with grade > 2 CRS [20]. Fever and usage of tocilizumab were therefore more frequent in patients with CTRCD (see also Table 1). As for CV events, we recorded only one case of non-fatal cardiac arrest in the context of severe CRS, a case of acute heart failure and a case of paroxysmal atrial fibrillation.

### Patterns of CTRCD

**Fig. 2:**
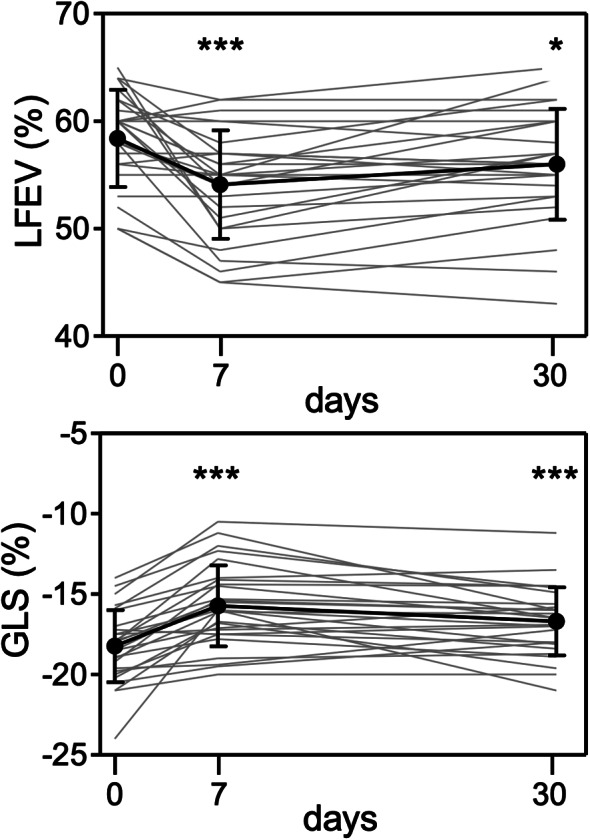
Temporal trends of LVEF and GLS in patients treated with CAR-T cells. Each panel shows individual data and means ± SD (at baseline and 7 and 30 days after CAR-T cells). Single or triple asterisks indicate *P* < 0.05 or *P* < 0.001 for LVEF and GLS at day 7 or 30 versus baseline. Abbreviations GLS = global longitudinal strain; LVEF = left ventricular ejection fraction

**Fig. 3:**
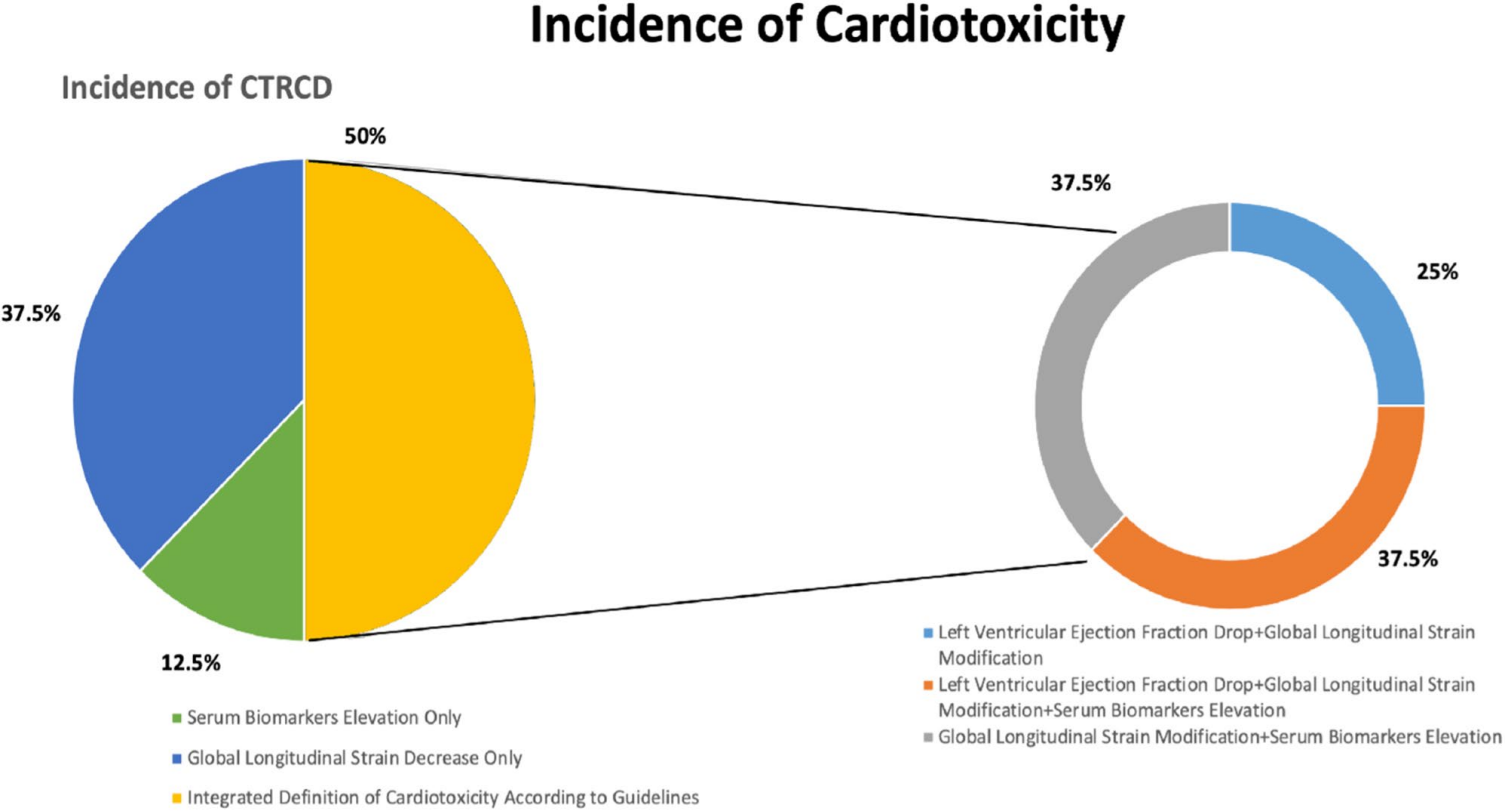
Graphical distribution of cancer therapy-related cardiac dysfunction (CTRCD) according to the definition used

Out of 16 patients diagnosed with CTRCD, 2 (12,5%) were characterized by only elevations of hsTnI or NT-proBNP, 6 (37,5%) were characterized by GLS decline ≥ 15% from baseline, and 8 (50%) were characterized by a composite of biomarkers and GLS with or without LVEF decrements (Fig. 2). Overall, GLS decrements were observed in 14 of 16 cardiotoxicity cases, followed by serum biomarkers elevations and LVEF decrements (9 and 5 of 16 cases, respectively). Patterns of LVEF and GLS changes are shown in Fig. 3, while a complete description of echocardiographic findings is reported in Table 1, Supplementary Materials.

**Table 1:** Patient baseline characteristics (overall and according to the occurrence of cancer therapy-related cardiac dysfunction)

	Overall population 27 patients (100%)	CTRCD 16 patients (59,3%)	No CTRCD 11 patients (40,7%)	P value
Demographics				
Age, years [mean ± standard deviation]	60.5 ± 10.4	58.8 ± 11.7	63.1 ± 7.9	0.260
Female sex [n, (%)]	9 (33.3)	8 (88)	1 [11]	0.042
**Cardiovascular risk factors**				
Hypertension [n, (%)]	20 (74.1)	10 (62.5)	10 (90.1)	0.183
Diabetes [n, (%)]	4 (14.8)	2 (12.5)	2 (18.2)	1,000
Smoking [n, (%)]	12 (44.4)	6 (37.5)	6 (54.5)	0,452
Dyslipidemia [n, (%)]	4 (14.8)	3 (18.8)	2 (18.2)	1,000
Previous history of IHD [n, (%)]	0 (0)	0 (0)	0 (0)	
**Electrocardiographic parameters**				
Heart rate, bpm [mean ± standard deviation]	84.1 ± 16.5	86.1 ± 13.6	80.5 ± 21.8	0.586
PR duration, milliseconds [mean ± standard deviation]	156.0 ± 32.8	155.0 ± 29.6	141.0 ± 41.6	0.939
QRS duration, milliseconds [mean ± standard deviation]	91.6 ± 8.6	90.7 ± 8.0	93.5 ± 10.2	0.567
Complete left bundle branch block, [n, (%)]	0 (0)	0 (0)	0 (0)	
Complete right bundle branch block, [n, (%)]	0 (0)	0 (0)	0 (0)	
**Medications**				
Beta-blockers [n, (%)]	8 (29.6)	4 (25.0)	4 (36.4)	0.675
ACE inhibitors/ARBs	10 (37.0)	5 (31.3)	5 (45.5)	0.687
MRA [n, (%)]	0 (0)	0 (0)	0 (0)	
**Prior Oncologic Therapies** Anthracycline cumulative dose, mg/m2 [median (IQR)]	585.0(566.0-1019.0)	579.0 (561.0-888.0)	600.0 (574.0-1034.0)	0.562
Previous Autologous HSCT [n, (%)]	14 (51.9%)	9 (56.3)	5 (45.5)	0.704
Number of Previous Chemotherapy Lines mean ± standard deviation]	2.79 ± 1.29	2.94 ± 1.61	2.55 ± 0.69	0.456
**CAR-T cell formulation**				
Axicabtagene Ciloleucel [n, (%)]	10 (37.1)	7 (43.8)	3 (27.3)	0.448
Tisagenlecleucel [n, (%)]	9 (33.3)	4 (25.9)	5 (45.4)	0.411
Brexucabtagene Autoleucel [n, (%)]	8 (29.6)	5 (31.3)	3 (27.3)	1.000
**Laboratory data**				
PLT x10^3^/L [median (IQR)]	154.0 (97.0-186.5)	157.0(100.5-216.3)	137.0(95.5–157)	0.267
Serum creatinine, mg/dL [mean ± standard deviation]	0.9 ± 0.3	0.9 ± 0.3	1.0 ± 0.2	0.320
Fibrinogen, mg/dL [median (IQR)]	387.0 (297.0-466.0)	409.0 (296.0-556.0)	374.0 (310.0-387.0)	0.208
D-Dimer [median (IQR)]	997.0 (754.0-1573.0)	1078.0 (751.0-1804.0)	997.0 (915.0-1323.0)	0.981
Ferritin, ng/mL [median (IQR)]	193.0 (129.0-479.0)	195.0 (134.0-531.0)	193.0 (132.0-479.0)	0.981
Partial thromboplastin time, seconds [mean ± standard deviation]	30.3 ± 4.4	29.7 ± 4.6	31.3 ± 4.2	0.364
Antithrombin-III, % [mean ± standard deviation]	99.0 ± 11.5	97.5 ± 11.3	101.0 ± 11.9	0.431
VIII Factor, % [median (IQR)]	193.0 (154.0-225.0)	195.0 (157.0-223.0)	193.0 (150.0-221.0)	1.000
Von Willebrand Factor, % [median (IQR)]	207.0 (180.0-260.0)	207.0 (182.0-253.0)	220.0 (177.0-284.0)	0.827
**Cardiac biomarkers**				
hs-Troponin T, ng/mL [median (IQR)]	5.0 (4.0–6.0)	5.0 (3.8-5.0)	5.0 (5.0–7.0)	0.179
NT-proBNP, pg/mL [median (IQR)]	30.5 (15.5–124.0)	57.0 (22.0-142.0)	16.0 (12.0-111.0)	0.121
sST2, ng/mL [median (IQR)]	27.0 (21.5–42.8)	27.0 (18.5–36.3)	27.0 (22.3–52.8)	0.470
**Inflammatory biomarkers**				
Interleukin 2 receptor, UI/L [median (IQR)]	1416.0 (1113.0-2378.0)	1334.0 (1160.0-1930.0)	1540.0 (1084.0-2378.0)	0.923
Interleukin 6, ng/L [median (IQR)]	23.6 (12.3–86.9)	31.9 (11.3–155.0)	19.6 (13.4–28.6)	0.633
**Echocardiographic parameters**				
LVEDV, ml [mean ± standard deviation]	94.5 ± 19.5	91.6 ± 21.4	98.6 ± 16.3	0.344
LVESV, ml [median (IQR)]	39.0 (30.0-47.5)	34.5 (29.0-44.5)	40 (35.5–47.5)	0.387
LV Simpson Biplane EF, % [median (IQR)]	60.0 (56.0-61.5)	60.0 (55.0-62.3)	60.0 (56.5–60.0)	0.765
LV GLS, % [mean ± standard deviation]	-18.2 ± 2.2	-17.9 ± 2.6	-18.8 ± 1.6	0.260
Medial mitral S’ velocity, cm/s [mean ± standard deviation]	9.4 ± 1.8	9.1 ± 2.0	9.9 ± 1.4	0.276
E/A ratio [median (IQR)]	0.8 (0.7-1.0)	0.8 (0.7-1.0)	0.8 (0.7–1.1)	0.708
E/e’ ratio, units [median (IQR)]	6.0 (5.0-8.5)	5.5 (4.0–7.0)	9.0 (6.0-10.5)	0.011
LAV max, ml [mean ± standard deviation]	47.6 ± 12.6	44.9 ± 12.5	51.4 ± 12.2	0.197
LA Reservoir Strain, % [mean ± standard deviation]	22.3 ± 5.4	21.6 ± 6.3	23.4 ± 4.1	0.392
TAPSE, mm [median (IQR)]	21.0 (18.0-23.5)	20.5 (18.0–23.0)	22.0 (20.0-24.5)	0.485
S’ RV, cm/s [mean ± standard deviation]	12.8 ± 2.4	12.4 ± 2.4	13.3 ± 2.5	0.398
**Cytokine release syndrome**	24 (88,8)	16 (100)	8 (72.7)	0.057
Cytokine Release Syndrome grade ≥ 2, [n, (%)]	19 (70.4)	15 (93.8)	4 (36.4)	0.002
Neurotoxicity, [n, (%)]	10 (37.0)	5 (31.3)	5 (45.5)	0.687
Fever, [n, (%)]	23 (85.2)	16 (100)	7 (63.6)	0.019
Hypotension, [n, (%)]	14 (51.8)	10 (62.5)	4 (36.4)	0.252
Tocilizumab Use, [n, (%)]	19 (70.4)	15 (93.8)	4 (36.4)	0.002
Corticosteroid Use, [n, (%)]	10 (37.0)	6 (37.5)	4 (36.4)	1.000

CAR-T: chimeric antigen receptor- T cells; **CTRCD =** cancer therapy-related cardiac dysfunction; CVD = cardiovascular disease; EDV = end-diastolic volume; EF = ejection fraction; ESV = end-systolic volume; GLS = global longitudinal strain; Hb = haemoglobin; HSCT = hematopoietic stem cell transplantation; IQR = interquartile range; LA = left atrial; LAEF = Left Atrial Emptying Fraction; LAV = left atrial volume; LV = left ventricular; MRA = mineralocorticoid receptor antagonist; NT-proBNP = N-terminal pro-brain natriuretic peptide; PLTs = platelets; RV = right ventricle; TAPSE = tricuspid annulus plane systolic excursion; WBC = white blood cells

**Fig. 4:**
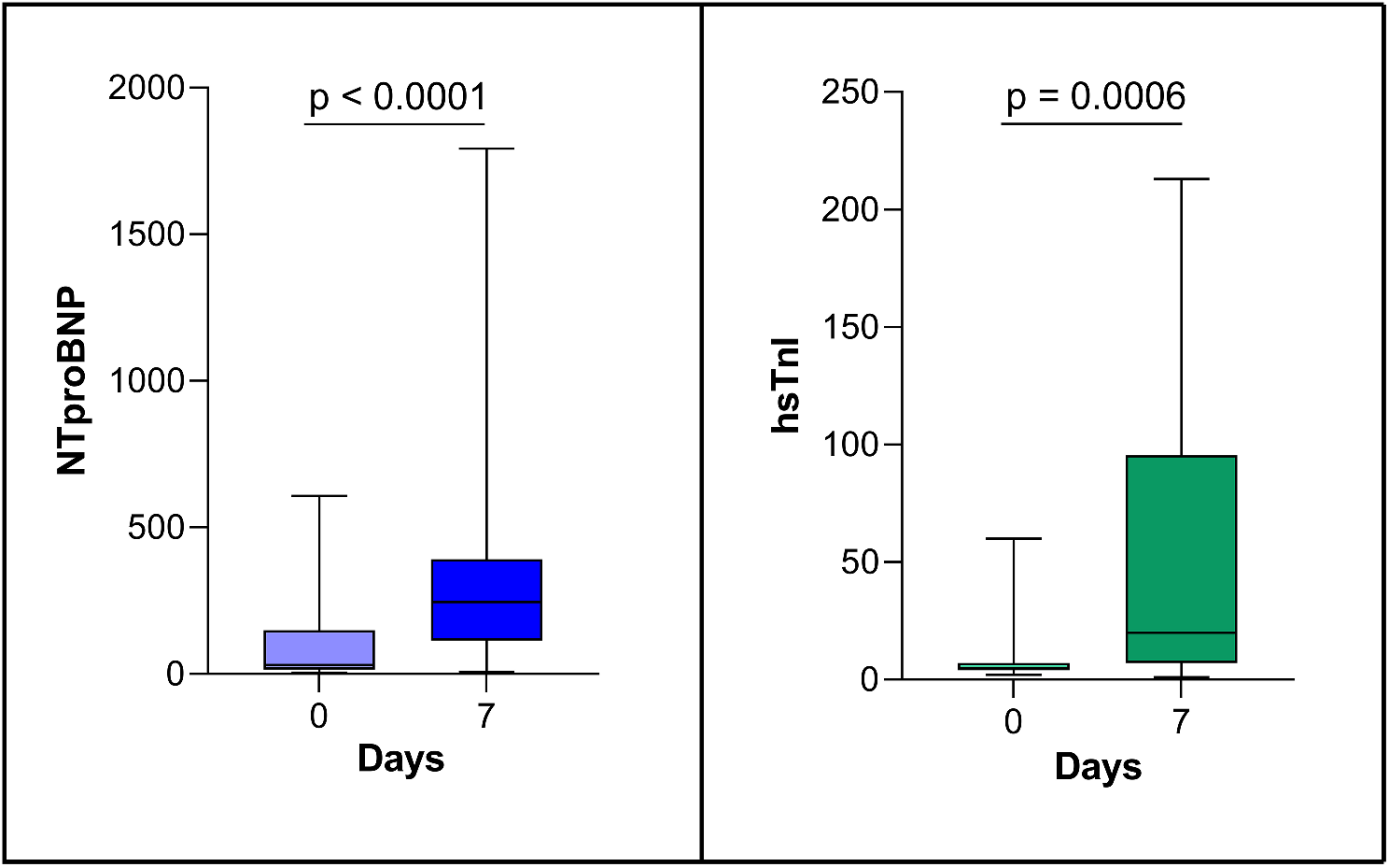
Early modifications (7 days after infusion to baseline) of serum cardiac biomarkers. Abbreviations hsTnI = high sensitivity troponin I; NTproBNP = N-terminal pro b-type natriuretic peptide

Changes in serum cardiac biomarkers at day 7 after CAR-T cell therapy are reported in Table 2; Fig. 4. In addition to significant elevations of hsTnI and NT-proBNP, there was a significant increase of sST2, marker of myocardial fibrosis (see also Table 2).

**Table 2:** Temporal Trends of biomarkers evaluated at two timepoints (baseline before chimeric antigen receptor-T cell infusion and 7 days after administration)

	Baseline	7 days	p-value	
Cardiac Biomarkers				
hs-Troponin T, ng/mL [median (IQR)]	5.0 (4.0–6.0)	20.0 (7.0-94.5)	*< 0,001*	
NT-proBNP, pg/mL [median (IQR)]	30.5 (15.5–124.0)	245.0 (152.0–363.0)	*< 0,001*	
sST2, ng/mL [median (IQR)]	27.0 (21.5–42.8)	54.0 (24.5–140.0)	*0.032*	

hs = high sensitivity; NT-proBNP = N-terminal prohormone of brain natriuretic peptide; sST2 = soluble ST2

### Correlations between left ventricular systolic function and inflammatory markers

**Fig. 5:**
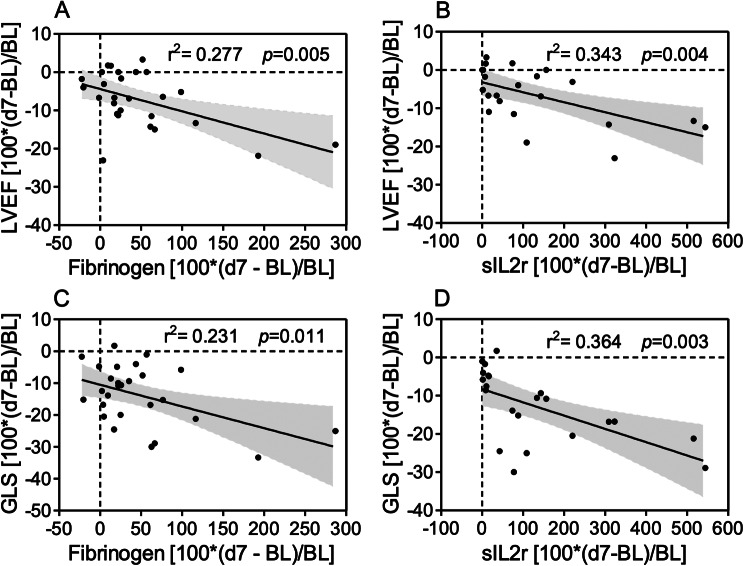
Significant Inverse Correlations between Early Changes in Echocardiographic Parameters and Inflammatory Indexes. Data were linear regression analyses with 95% confidence intervals of percentage differences of echocardiographic parameters versus inflammatory indexes, all expressed as 100*(day 7 minus baseline)/baseline. Panel **A**, LVEF versus fibrinogen; Panel **B**, LVEF versus sIL2r; panel **C**, GLS versus fibrinogen; panel **D**, GLS versus sIL2r. Similar results were obtained by non-parametric two-tailed correlation (*p* = 0.052, 0.005, 0.062 and 0.0001 in panels **A**, **B**, **C** and **D**, respectively). Abbreviations GLS = left ventricular global longitudinal strain; sIL2r = soluble interleukin 2 receptor; LVEF = left ventricular ejection fraction; d7 = day 7; BL, baseline

We characterized whether day 7 changes of LVEF and GLS, parameters of systolic function reported in ESC guidelines definition of CTRCD [13], correlated with changes of inflammatory biomarkers at the same time point. A statistically significant inverse correlation occurred between changes of LVEF or GLS and sIL2r or fibrinogen (Fig. 5). No correlation was observed with CRP, IL6, ferritin (not shown).

## Discussion

Uncertainties remain around the actual incidence of CTRCD in patients treated with CAR-T cells [21–27]. A study of 137 patients found that 5.8% of them developed a significant drop in LVEF (defined as a decrease of at least 10% points to a value below 50%), mainly associated with the occurrence of grade ≥ 2 CRS [22]. Other studies showed that a reduction of LVEF below 50% or > 10% from baseline during index hospitalization occurred in 10.3% of 116 patients, with a decline in median LVEF from 58 to 37% at ∼ 12 days from CAR T-cell infusion [23]. Again, most of patients diagnosed with CTRCD had grade ≥ 2 CRS, further highlighting possible cause-and-effect relations between systemic inflammation and cardiotoxicity. On the other hand, the recently released ESC Cardio-Oncology guidelines recommend a definition of CTRCD that integrates abnormalities of imaging parameters and serum biomarkers, such as LVEF, GLS, troponin and natriuretic peptide [13]. In accordance with this definition, we were able to diagnose CTRCD in as many as 16 patients treated with CAR-T cells. Had we defined CTRCD only on the basis of LVEF decrements, its incidence would have been 18,5% (5 cases out of 27 patients).

The high incidence of CTRCD observed in our study warrants further considerations. Our patients received serial echocardiographic and biomarkers evaluations, which were done prospectively at pre-specified time points. This approach likely avoided the risk of underestimating CTRCD, that otherwise would bias studies in which imaging and laboratory evaluations were event-driven. In this context, it was not until recently when Lefebvre et al. prospectively evaluated 44 patients prior to and 2 days, 1 week, 1 month, and 6 months after CAR-T cell infusion [11]. No significant changes in LVEF were noticed across study visits and only a modest decrease in GLS was shown to occur at the early time points. Interestingly, only 52% of subjects developed CRS as opposed to at least 70% in previous reports and 89% in our present study; moreover, CRS was of a low grade in 95% of cases [11]. Lower incidence and severity of CRS may help to explain the milder cardiovascular manifestations observed by Lefebvre et al. compared to our study.

Having said that the high incidence of CTRCD observed in our study originates from the updated definition of CTRCD released by ESC [13], largely based on subclinical indexes like GLS and serum biomarkers, we believe that detecting such early manifestations might be important for improving CV outcomes in the cancer patient. A multicenter registry of 202 CART-cell patients receiving anti-CD19 therapies aimed at assessing a composite endpoint of heart failure, cardiogenic shock, or myocardial infarction [25]. Sixteen percent of subjects experienced severe cardiac events, which were independently associated with overall mortality (hazard ratio 2.8). In analyzing determinants of event occurrence the authors identified a role for CV risk factors, in particular hypertension and history of atrial fibrillation or heart failure; interestingly, however, there was no information on clinical usage of anti-IL6 medications to mitigate CRS in these patients [25]. Post-marketing analyses similarly showed a fatality rate of CV and pulmonary adverse events, including late-occurring cardiomyopathy, tachyarrhythmias, pleural and pericardial effusions, as high as 30.9% [26]. None of our patients presented at treatment with a history of heart failure, ischemic heart disease, or arrhythmias, which probably explains why we did not record a significant incidence of fatal CV events after CAR-T cell therapy. However, the high incidence of CTRCD that we characterized as early as 7 days after CAR-T cell infusion, serves a rationale to intensify CV surveillance in high-risk patients with a potentially worse CV outcome. The ESC cardio-oncology guidelines advocate intensive surveillance in cases of signs and symptoms of congestion or high-grade CRS, but they do not clarify how often and how long patients should be surveilled [13].

There are further differences, as well as similarities, between our study and previously published reports. Older age, dyslipidemia and coronary artery disease were reported to increase the risk of cardiomyopathy after CAR-T cell therapy [22]. As already mentioned, our study did not recruit patients with a history of ischemic heart disease but hypertension, diabetes and smoking were equally represented among patients with or without CTRCD. On a different note, but in agreement with others [22, 23], we also found that neither the number of prior lines of therapy nor the cumulative anthracycline dose were significantly different among patients with or without CTRCD. This latter finding denotes the distinct nature of CAR-T cells cardiotoxicity, as previous treatment with anthracyclines usually aggravates the risk of cardiotoxicity upon patient’s exposure to subsequent cancer therapies.

### Limitations

We acknowledge this was a single institution study, with a small sample size. The lack of a control arm in which patients with the same oncologic diagnosis received treatments with other drugs, possibly including newly developed bispecific antibodies that also cause some degree of CRS, preclude further considerations on the actual risk:benefit of CAR-T cells in terms of CV liability. Furthermore, the very low incidence of major CV events in our study population, likely reflecting the extensive use of anti-IL6 medication and short-follow-up, does not allow us to approximate how well such events would have been predicted by the imaging and bio-humoral markers we used to define CTRCD. Finally, the majority of subjects enrolled were males, preventing generalization of findings to females.

### Strengths

In addition to denoting the value of integrating echocardiographic parameters with serum biomarkers, this study provides novel information on the relations between inflammation and CTRCD. We in fact confirmed a higher incidence of CTRCD in patients with grade ≥ 2 CRS, but we also investigated, for the first time in adult patients [27], on the association between changes in LVEF or GLS and inflammatory biomarkers. We did not find correlations with CRP, IL6 and ferritin, as tocilizumab interferes with IL6 assay [28] and reduces both CRP [29] and ferritin levels [30]; however, we found significant correlations with sIL2R, whose levels are relatively stable after tocilizumab initiation [31], and with fibrinogen, which shows longer half-life than ferritin [32, 33] and thus attains circulating levels more suitable for correlation analyses once tocilizumab has been started. These findings strengthen a causative link between inflammation and CAR-T cells cardiotoxicity, paving the road to further studies in these settings [34].

In addition, besides conventional cardiac biomarkers such as hsTnI and NT-proBNP, we characterized early changes of sST2, currently considered as an index of myocardial remodeling and fibrosis [35]. sST2 significantly increased at day 7, similar to hsTnI and Nt-proBNP (Table 2). As the ST2 gene is upregulated in the setting of myocardial stretch, these findings raise one more research issue in the settings of CAR-T cells cardiotoxicity [35].

## Conclusions

This prospective study shows, for the first time, that a large proportion of patients treated with anti CD19 CAR-T cells may experience acute CTRCD, as defined by recent Cardio-Oncology guidelines. Moreover, a remarkable association of CTRCD with an inflammatory *primum movens* is confirmed by more direct correlations than in previous studies [36]. We therefore propose a systematic approach of clinical surveillance and comprehensive evaluation of patients undergoing CAR-T cell therapy, including both imaging and laboratory indexes as suggested by cardio-oncology guidelines. Early monitoring would remarkably assist the identification of patients at risk of developing severe cardiomyopathy. An extended follow-up, which was beyond the aims of this proof-of-concept study, would in turn elucidate the size effect of acute cardiotoxicity on late clinical outcomes.

### Electronic supplementary material

Below is the link to the electronic supplementary material.

Supplementary Material 1

## Data Availability

The datasets used and analysed in this study are available from the corresponding author on reasonable request.

## Abbreviations

CAR-T: chimeric antigen receptor-T
CRP: c-reactive protein
CRS: cytokine release syndrome
CTRCD: cancer therapy-related cardiac dysfunction
CV: cardiovascular
IL: interleukin
LA: left atrium
LVEF: left ventricular ejection fraction
LVGLS: left ventricular global longitudinal strain
