# Supplementary material for: Inflammation and acute cardiotoxicity in adult hematological patients treated with CAR-T cells: results from a pilot proof-of-concept study
Source: Cardiooncology. 2024 Mar 27;10:18. doi: 10.1186/s40959-024-00218-0 (PMC10967131; doi:10.1186/s40959-024-00218-0)
Supplement: Supplementary file 1 — Supplementary Material 1 [file 40959_2024_218_MOESM1_ESM.docx]

**Table 3. Temporal Trends of echocardiographic metrics evaluated at three timepoints (baseline and 7 and 30 days after CAR-T cells).**

|  | **Baseline** | **7 days** | ***p-value 0-7*** | | **30 days** | ***p-value 7-30*** | ***p-value 0-30*** |  |  |
| --- | --- | --- | --- | --- | --- | --- | --- | --- | --- |
| **Echocardiographic parameters**  *Left Ventricular Dimensions and Function* | | | |  |  |  |  |  |  |
| LVEDV, ml/m [mean ± SD] | 94.5±19.5 | 92.4±19.5 | | *0,6327* | 90.7 ± 19.2 | *0,6793* | *0.3820* |  |  |
| Simpson Biplane LVEF, % [median (IQR)] | 60.0 (56.0-61.5) | 55.0 (50.5-57.0) | | *0.0001* | 56.0 (53.5-60.0) | *0.0089* | *0.0287* |  |  |
| LV GLS, % [mean ± SD]    *Left Ventricular Diastolic Function* | -18.2±2.2 | -15.7±2.5 | | *<0,0001* | -16.7 ± 2.1 | *0,0247* | *<0,0001* |  |  |
| E/A ratio, units [median (IQR)] | 0.8 (0.7-1.0) | 0.8 (0.7-0.9) | | *0,5349* | 0.8 (0.6-1.0) | *0,8765* | *0.4384* |  |  |
| E/e’ ratio, units [median (IQR)] | 6.0 (5.0-8.5) | 8.0 (6.2-9.5) | | *0,0046* | 8.0 (7.0-9.5) | *0.6743* | *0.0014* |  |  |
| *Atrial Structure and Function* | |  | |  |  |  |  |  |  |
| LAV max, ml/m [mean ± SD] | 47.6 ± 12.6 | 54.1±13.7 | | *0,0024* | 49.6 ± 12.7 | *0,0234* | *0.3549* |  |  |
| LA Reservoir Strain, % [mean ± SD] | 22.3±5.5 | 17.8±4.4 | | *<0,0001* | 20.7 ± 6.5 | *0,0409* | *0,4452* |  |  |
|  |  |  | |  |  |  |  |  |  |

**Legend:** EDV = end-diastolic volume; EF = ejection fraction; ESV = end-systolic volume; GLS = global longitudinal strain; LA = left atrial; LAV = left atrial volume; LV = left ventricular
